# The molecular pathology of schizophrenia: an overview of existing knowledge and new directions for future research

**DOI:** 10.1038/s41380-023-02005-2

**Published:** 2023-03-06

**Authors:** Takumi Nakamura, Atsushi Takata

**Affiliations:** 1 Laboratory for Molecular Pathology of Psychiatric Disorders, RIKEN Center for Brain Science, 2-1 Hirosawa, Wako, Saitama 351-0198 Japan; 2 Research Institute for Diseases of Old Age, Juntendo University Graduate School of Medicine, 2-1-1 Hongo, Bunkyo-Ku, Tokyo, 113-8421 Japan

**Keywords:** Schizophrenia, Genetics, Neuroscience, Molecular biology

## Abstract

Despite enormous efforts employing various approaches, the molecular pathology in the schizophrenia brain remains elusive. On the other hand, the knowledge of the association between the disease risk and changes in the DNA sequences, in other words, our understanding of the genetic pathology of schizophrenia, has dramatically improved over the past two decades. As the consequence, now we can explain more than 20% of the liability to schizophrenia by considering all analyzable common genetic variants including those with weak or no statistically significant association. Also, a large-scale exome sequencing study identified single genes whose rare mutations substantially increase the risk for schizophrenia, of which six genes (*SETD1A*, *CUL1*, *XPO7*, *GRIA3*, *GRIN2A*, and *RB1CC1*) showed odds ratios larger than ten. Based on these findings together with the preceding discovery of copy number variants (CNVs) with similarly large effect sizes, multiple disease models with high etiological validity have been generated and analyzed. Studies of the brains of these models, as well as transcriptomic and epigenomic analyses of patient postmortem tissues, have provided new insights into the molecular pathology of schizophrenia. In this review, we overview the current knowledge acquired from these studies, their limitations, and directions for future research that may redefine schizophrenia based on biological alterations in the responsible organ rather than operationalized criteria.

## Introduction

The term pathology is defined as the study of the essential nature of disease [1]. In the pathology of physical diseases, abnormal changes in responsible organs or systems, such as invasion of malignant cells in cancers, and loss of nigrostriatal dopaminergic neurons in Parkinson’s disease, are examined typically by microscopes. On the other hand, a field of study of pathological cognition and behaviors often observed in patients with psychiatric disorders, psychopathology, does not analyze the brain, the organ presumably responsible for these disorders. This may not be unreasonable given that psychiatry is etymologically a study of the psyche, a Greek word ψυχή whose derived meaning includes invisible spirit or soul. Nevertheless, It has long been thought that mental illness is fundamentally a disease of the brain, as classically advocated by Wilhelm Griesinger, Emil Kraepelin, and others [2, 3]. Based on this concept, the brain pathology of schizophrenia, a common psychiatric disorder with a lifetime prevalence of ~1% [4], has been investigated in numerous studies. However, despite many efforts, no definite pathological changes, like senile plaques and neurofibrillary tangles in Alzheimer’s disease, were identified in their postmortem brains. Meanwhile, recent rapid advances in molecular biology and engineering have prompted the development of methods to analyze molecules such as nucleotides and proteins more comprehensively, more sensitively, with a higher cellular and spatial resolution, and quantitatively. Besides, multiple collaborative research frameworks have been established to ensure sample sizes sufficient to solve the critical problem of multiple testing and relevant statistical power. This has especially been true in comprehensive studies of variants in DNA, which encodes fundamental biological information for all organs, including the brain and can be analyzed by using easily accessible peripheral tissue samples, resulting in an accumulation of statistically robust findings. Given these, while we should refrain from being overly optimistic, perhaps now might be the time to bring together these technologies and resources to elucidate the molecular pathology of schizophrenia. In this review, we overview the findings from research aiming to decipher the molecular pathology of schizophrenia, emphasizing large-scale omics studies with substantial statistical power and analyses of etiologically valid disease models, and summarize the current achievements. Following that, the challenges and obstacles to be overcome and the future research directions will be discussed, along with an introduction of preliminary results from studies utilizing cutting-edge technologies that will surely facilitate the elucidation of the fundamental pathology of schizophrenia.

## The genetic pathology of schizophrenia

Almost unquestionably, the brain is the organ primarily responsible for the pathogenesis of schizophrenia. However, the human brain is covered by thick cranial bones, and therefore it is almost impossible to access and analyze living human brain tissues at the molecular level in a non-invasive manner, nor is it easy to collect postmortem brains on a large scale. On the other hand, the sequences of DNA, the molecule encoding fundamental biological information of all tissues, can be analyzed without accessing the brain, since its sequences are in principle identical in every cell and invariant throughout life, with few exceptions. Based on this relative ease in obtaining samples as well as the high heritability of schizophrenia, reported to be 60–80% in epidemiological studies [5–8], large-scale genetic studies, analyzing samples from more than 100,000 individuals these days, have been conducted. Reflecting this large number, among various research on the molecular pathology of schizophrenia, statistically robust findings have been particularly accumulated from human genetics studies analyzing variations of the sequences of DNA molecules. For this reason, we begin with an overview of our knowledge of the genetic pathology of schizophrenia.

### Robust findings from large-scale analyses of common and rare variants

Although schizophrenia is a highly heritable disorder, no single variant explaining a large portion of the overall heritability has been identified. Therefore, comprehensive studies of various allele frequencies and effect sizes of genetic variants contributing to its risk are warranted. In a simplified view, there are two major types of genetic studies on different frequencies and effect sizes of variants, that is, genome-wide association studies (GWAS) of common single nucleotide polymorphisms (SNPs) with small effect sizes (typically odds ratio [OR] < 1.2) and sequencing studies of rare variants potentially with large effect sizes (sometimes OR > 10). The scales of these two types of studies have consistently grown [9–19], and the results of the two largest studies to date, each looking into common SNPs [20] or rare variants [21], have recently been published after peer review.

In the newest GWAS of common SNPs by the Psychiatric Genomics Consortium (PGC) analyzing 76,755 schizophrenia cases and 243,649 control individuals [20], 287 distinct loci with genome-wide significant association (*P* < 5 × 10^−8^) were identified. The SNP with the largest effect size was the rs140365013 variant on chromosome 6 near the major histocompatibility complex region, with an OR of 1.23, confirming that individual SNPs do not greatly increase the disease risk. On the other hand, the proportion of the variance in schizophrenia liability explained by all measured SNPs, including numerous variants that did not show statistically significant association with schizophrenia, was reported to be 24%. This proportion is much larger than that calculated solely from loci associated with genome-wide significance. Therefore, a significant part of the heritability is attributable to common SNPs that individually show weak associations and effects, and it can be said that schizophrenia is a highly polygenic disorder. Since each of the 287 associated loci often contains multiple genes (specifically, of the 287 loci, 206 and 108 contain ≥2 and ≥5 genes, respectively), it is essential to identify functionally important causal genes and variants in order to understand the molecular pathology from the genetic pathology. To this end, gene prioritization was performed in this GWAS by PGC using various approaches such as fine-mapping of credible sets of causal SNPs by an adaptation of a Bayesian inference algorithm [22] and Mendelian randomization to identify SNPs whose causal effects are likely to be mediated through regulation of gene expression [23]. As a result, a total of 120 prioritized genes were nominated, of which 70 and 55 received support from the fine-mapping and Mendelian randomization analyses, respectively. There were five genes supported by both lines of evidence (*CUL9*, *FURIN*, *LINC00320*, *SNAP91*, and *ZNF823*). In addition, two other prioritized genes (*GRIN2A*, and *SP4*) are supported by statistical evidence significant after conservative multiple testing correction in the rare variant study described below (Table 1a, b).

**Table 1:** Prioritized schizophrenia genes from common and rare variant studies.

*a. PGC GWAS of Common SNPs (76,755 cases and 243,649 controls, ref*. [20])*														
Gene Symbol	Ensembl gene ID	Chromosome	Gene biotype	Index SNP ID	Position (hg19)	Ref	Alt	Uncorrected P value	Control frequency	PGC odds ratio (95% CI)	Prioritized by fine mapping	Prioritized by Mendelian randomization	SCHEMA uncorrected P value	SCHEMA odds ratio** (95% CI)
*CUL9*	ENSG00000112659	6	protein_coding	rs113113059	43160375	T	C	2.29E-11	0.756	1.06 (1.04-1.08)	Yes	Yes	0.548	1.03 (0.436–2.19)
*FURIN*	ENSG00000140564	15	protein_coding	rs4702	91426560	G	A	2.15E-23	0.446	1.08 (1.07-1.10)	Yes	Yes	0.066	2.68 (0.555–11.3)
*GRIN2A*	ENSG00000183454	16	protein_coding	rs9926049	9939960	C	A	3.16E-10	0.736	0.95 (0.93-0.96)	Yes	No	7.37E-07^†^	24.1 (5.36-221)
*LINC00320*	ENSG00000224924	21	lincRNA	rs459391	22120508	T	C	1.54E-08	0.185	1.06 (1.04-1.08)	Yes	Yes	NA	NA
*SNAP91*	ENSG00000065609	6	protein_coding	rs2022265	84293271	A	G	3.74E-10	0.523	1.05 (1.03-1.07)	Yes	Yes	1	0 (0–6.08)
*SP4*	ENSG00000105866	7	protein_coding	rs7811417	21534152	T	C	2.17E-09	0.347	1.05 (1.03-1.07)	Yes	No	5.08E-07^†^	7.59 (3.2-19.3)
*ZNF823*	ENSG00000197933	19	protein_coding	rs72986630	11849736	C	T	3.07E-10	0.939	0.89 (0.86-0.93)	Yes	Yes	0.438	1.38 (0.602–2.92)

*b. SCHEMA study of rare SNVs/indels (24,248 cases, 97,322 controls, and 3,402 case trios, ref*. [21]*)*												
Gene Symbol	Ensembl gene ID	Chromosome	Case LOF/PTV count	Control LOF/PTV count	Case missense (MPC ≥ 3) count	Control missense (MPC ≥ 3) count	De novo LOF/PTV count	Uncorrected P	Bonferroni	Odds ratio* (95% CI)	OMIM gene ID	OMIM phenotype
*SETD1A*	ENSG00000099381	16	15	3	3	4	3	2.00E-12	4.66E-08	10.3 (4.12-29.3)	611052	Neurodevelopmental disorder with speech impairment and dysmorphic facies
*CUL1*	ENSG00000055130	7	8	1	2	0	3	2.01E-09	4.69E-05	44.2 (6.42-253)	603134	None
*XPO7*	ENSG00000130227	8	12	1	1	1	1	7.18E-09	0.000167	28.1 (6.46-253)	606140	None
*TRIO*	ENSG00000038382	5	18	16	0	0	2	6.35E-08	0.001481	5.02 (2.47-10.4)	601893	Intellectual developmental disorder, autosomal dominant, with microcephaly
*CACNA1G*	ENSG00000006283	17	10	13	8	4	0	4.57E-07	0.010658	4.25 (2.07-8.78)	604065	Spinocerebellar ataxia, early-onset, severe, with neurodevelopmental deficits
*SP4*	ENSG00000105866	7	13	6	3	3	1	5.08E-07	0.011847	7.59 (3.2-19.3)	600540	None
*GRIA3*	ENSG00000125675	X	5	0	3	2	1	5.98E-07	0.013946	20.1 (4.28-188)	305915	Intellectual developmental disorder, X-linked, syndromic, Wu type
*GRIN2A*	ENSG00000183454	16	9	2	3	0	0	7.37E-07	0.017188	24.1 (5.36-221)	138253	Epilepsy, focal, with speech disorder and with or without impaired intellectual development
*HERC1*	ENSG00000103657	15	28	32	0	0	0	1.26E-06	0.029384	3.51 (2.04-6.03)	605109	None
*RB1CC1*	ENSG00000023287	8	9	4	0	0	2	0.000002	0.046642	10 (2.89-43.9)	606837	None

*c. PGC CNV Working Group study (21,094 cases and 20,227 controls, ref*. [31])									
Locus (gene)	Chromosome	Start (hg18)	End (hg18)	CNV type	Case count	Control count	Regional uncorrected P value	Odds ratio (95% CI)	Contained genes
22q11.21	22	17400000	19750000	Loss	64	1	5.70E-18	67.7 (9.3-492.8)	*DGCR2, AC004471.2, TSSK2, DGCR14, GSC2, SLC25A1, CLTCL1, HIRA, MRPL40, C22orf39, UFD1L, CDC45L, CLDN5, SEPT5, GP1BB, TBX1, GNB1L, C22orf29, TXNRD2, COMT, ARVCF, C22orf25, DGCR8, HTF9C, RANBP1, ZDHHC8, RTN4R, DGCR6L, AC007663.29, AC023490.5-2, GGTLC3, TMEM191C, PI4KAP1, RIMBP3, AC011718.2, AC007731.16-3, AC007731.16-4, USP41, ZNF74, SCARF2, KLHL22, MED15, POM121L4P, PI4KA, SERPIND1, SNAP29, CRKL, AC002470.17-2, AIFM3, LZTR1, THAP7, AC002472.8-1, P2RX6, SLC7A4, P2RX6P, AC002472.8-2*
16p11.2, proximal	16	29560000	30110000	Gain	70	7	2.52E-12	9.4 (4.2-20.9)	*SPN, QPRT, C16orf54, AC009133.7-1, KIF22, MAZ, PRRT2, C16orf53, MVP, CDIPT, AC120114.2-2, SEZ6L2, ASPHD1, KCTD13, TMEM219, TAOK2, HIRIP3, INO80E, DOC2A, AC093512.2, FAM57B, ALDOA, PPP4C, TBX6, YPEL3, GDPD3, MAPK3, CORO1A, BOLA2B, ZNF688, ZNF688*
2p16.3 (*NRXN1*)	2	50000992	51113178	Loss	35	3	4.92E-09	14.4 (4.2-46.9)	*NRXN1*
15q13.3	15	28920000	30270000	Loss	28	2	2.13E-07	15.6 (3.7-66.5)	*MTMR15, MTMR10, TRPM1, KLF13, OTUD7A, CHRNA7*
1q21.1	1	144646000	146176000	Loss + gain	60	14	1.50E-06	3.8 (2.1-6.9)	*PDZK1P2, PDZK1P2, NBPF8, NBPF8, FAM108A3, AL049742.8-2, NBPF12, PRKAB2, FMO5, CHD1L, BCL9, ACP6, GJA5, GJA8, GPR89B, PDZK1P2, NBPF11, NBPF11, FAM108A2, FAM108A2, BX842679.19*
3q29	3	197230000	198840000	Loss	16	0	1.86E-06	Infinity	*TFRC, ZDHHC19, AC069257.28-2, PCYT1A, TM4SF19, TCTEX1D2, UBXN7, C3orf43, RNF168, FBXO45, WDR53, C3orf34, LRRC33, PIGX, PAK2, SENP5, NCBP2, PIGZ, MFI2, DLG1, BDH1*
16p11.2, distal	16	28730000	28960000	Loss	11	1	5.52E-05	20.6 (2.6-162.2)	*ATXN2L, TUFM, SH2B1, ATP2A1, RABEP2, CD19, NFATC2IP, SPNS1, LAT, AC112166.3-2*
7q11.23	7	72380000	73780000	Gain	16	1	0.000168	16.1 (3.1-125.7)	*TRIM50, FKBP6, FZD9, BAZ1B, BCL7B, TBL2, MLXIPL, VPS37D, DNAJC30, WBSCR22, STX1A, ABHD11, CLDN3, AC093168.3, CLDN4, WBSCR27, WBSCR28, ELN, LIMK1, WBSCR1, LAT2, RFC2, CYLN2, GTF2IRD1, GTF2I*

*CI*: confidence interval; *FDR*: false discovery rate; *LOF/PTV*: loss-of-function/protein-truncating variant; *MPC*: missense badness, PolyPhen-2, and constraint; *SNP*: single nucleotide polymorphism.

*Results from the "core" meta-analysis downloaded from https://www.med.unc.edu/pgc/download-results/ are shown.

**Odds ratio for LOF/PTV and MPC ≥ 3 missense variants.

^†^Bonferroni-corrected P < 0.05.

*CI*: confidence interval; *LOF/PTV*: loss-of-function/protein-truncating variant; *OMIM*: Online Mendelian Inheritance in Man; *MPC*: missense badness, PolyPhen-2, and constraint; *SNV*: single nucleotide variant.

*Odds ratio for LOF/PTV and MPC ≥ 3 missense variants.

*CI*: confidence interval; *CNV*: copy number variant.

In a companion study of rare variants by the Schizophrenia Exome Meta-Analysis (SCHEMA) Consortium, rare single nucleotide variants (SNVs, any frequency of single nucleotide substitutions including both SNPs and rare variants) and short insertion/deletions (indels) in protein-coding regions were systematically analyzed by using exome (all protein-coding exonic regions) sequencing data of 24,248 schizophrenia cases, 97,322 controls, and 3402 trios consisting of schizophrenia probands and their unaffected parents. By evaluating the burden of rare loss-of-function (LOF: nonsense, splice site, and frameshift indel) variants (also known as protein-truncating variants: PTVs) and damaging missense variants (defined by the missense badness, PolyPhen-2, and constraint [MPC] score), including those arisen de novo in the probands, this study identified ten genes (*SETD1A*, *CUL1*, *XPO7*, *TRIO*, *CACNA1G*, *SP4*, *GRIA3*, *GRIN2A*, *HERC1*, and *RB1CC1*; Table 1b) surpassing the exome-wide significance threshold (defined as 2.14 × 10^−6^ based on the number of protein-coding genes analyzed) and 22 additional genes at a false discovery rate (FDR) < 0.05. Considering their effect sizes, six (*SETD1A*, *CUL1*, *XPO7*, *GRIA3*, *GRIN2A*, and *RB1CC1*) out of the ten exome-wide significant genes were enriched for rare PTV and damaging missense (MPC > 3) variants with OR > 10, indicating that rare deleterious variants of these genes confer the schizophrenia risk with large effects as well as robust statistical significance. Another notable thing is that five (*SETD1A*, *TRIO*, *CACNA1G, GRIA3*, and *GRIN2A*) of the ten genes are also implicated in other neurodevelopmental disorders, such as intellectual disability and epilepsy, as registered on the Online Mendelian Inheritance in Man (OMIM) database [24]. Therefore, on the one hand, it is possible that these cases with deleterious variants in known neurodevelopmental disorder genes might be individuals who should have been molecularly diagnosed as patients of highly heritable neurodevelopmental diseases, while on the other hand, another possibility is that the carriers of these variants did not exhibit developmental and physical symptoms sufficient to be diagnosed with a neurodevelopmental disease due to some modifying factors and were operationally diagnosed with schizophrenia. If the former is true, this suggests that genetic testing may detect overlooked patients with highly heritable neurodevelopmental diseases and provide clues for better intervention. If the latter is the case, it implies that some modifying factors influence the severity of the symptoms. Indeed, there is accumulating evidence supporting the existence of modifying factors, such as common and rare variants other than the diagnostic mutation, in patients with neurodevelopmental diseases [25–28]. A more detailed analysis of such modifying factors may pave the way toward the development of new treatment and prevention strategies.

Besides common SNPs analyzed in GWAS and rare SNVs/indels analyzed in exome sequencing studies, another important class of genetic variation of which several are known to be robustly associated with schizophrenia is copy number variants (CNVs) [29–32]. Among important works on CNVs, a large genome-wide study by the CNV Working Groups of PGC analyzing 21,094 schizophrenia cases and 20,227 controls [31] identified copy number losses at six loci (1q21.1, 2p16.3 involving *NRXN1*, 3q29, 15q13.3, distal 16p11.2, and 22q11.2) and gains at two loci (7q11.23 and proximal 16p11.2) that are significantly (*P* < 1.33 × 10^−4^ for losses and 4.33 × 10^−5^ for gains) associated with schizophrenia after multiple testing corrections (Table 1c). All of these are rare in controls and contribute to the risk for schizophrenia with ORs ranging from 3.8 to infinity. Also, like the known neurodevelopmental disorder genes identified in the SCHEMA study described above, all, or nearly all of these schizophrenia-associated CNVs are phenotypically pleiotropic and often more strongly associated with disorders other than schizophrenia, such as ASD and intellectual disability. Therefore, complex phenotype-genotype relationships should be considered when predicting disease risks from the information on CNVs and generating and analyzing CNV-based animal and cellular models.

Aggregating the findings from studies of common SNPs and rare SNVs/indels and CNVs, we can now explain a substantial part of the schizophrenia heritability (mainly by common SNPs) and have produced a list consisting of six genes and six CNVs associated with schizophrenia with observed ORs larger than ten. Also, there is a convergence of the results of studies of common and rare variants. At the level of individual genes, *GRIN2A* and *SP4* are included in the list of 120 genes prioritized in the PGC GWAS and showed exome-wide significant enrichment of rare deleterious variants in the SCHEMA study, as described above. At the level of overall enrichment patterns, the sets of genes implicated in the SCHEMA study and studies of rare coding variants in other neurodevelopmental disorders (e.g., ASD and intellectual disability) are shown to be significantly enriched for common variant associations in the PGC GWAS.

**Fig. 1:**
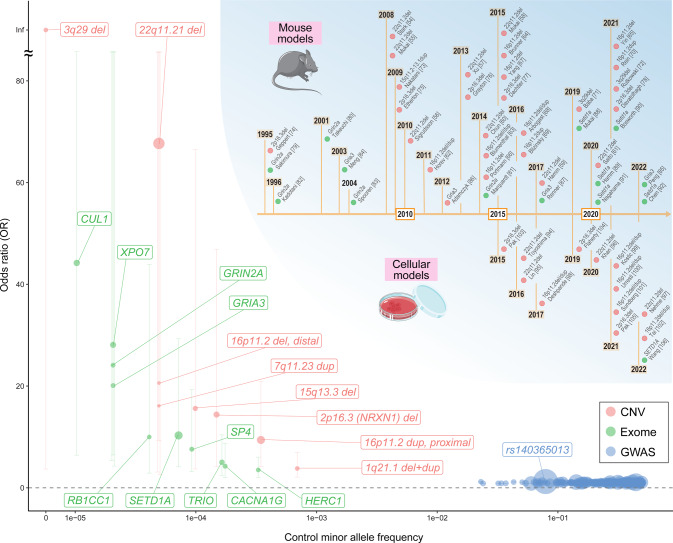
Allelic spectrum of schizophrenia-associated variants. A plot of minor allele frequencies in controls (*x*-axis) and ORs (y-axis) for schizophrenia-associated genes/variants with robust statistical evidence, that is, the index SNPs of 287 genome-wide significant loci identified by the PGC GWAS (blue, ref. [20]), ten exome-wide significant genes in the SCHEMA exome sequencing study (green, ref. [21]) and the study-wide significant eight CNVs in the PGC CNV Working Groups study (red, ref. [31]). Genes and variants with OR > 1.2 were labeled. The sizes of points are proportional to the −log_10_ *P* values for the association. The error bars indicate 95% confidence intervals of the ORs. The upper right chronology summarizes the representative studies of etiologically valid mouse and cellular models of schizophrenia shown in Tables 2 and 3.

We provide a compiled list of genes and CNVs identified through large-scale studies, which underlie the genetic pathology of schizophrenia, in Table 1, together with the landscape of their population frequencies and effect sizes in Fig. 1. Meanwhile, as shown in Table 1 and Fig. 1, it should be noted that there are wide ranges of confidence intervals for ORs for rare risk variants. Also, there is a possibility of the so-called winner’s curse [33] in genetic studies. To more accurately estimate their effect sizes and the robustness of the association, further larger studies are always warranted.

### Functional convergence of the findings in genetic studies

As described above, genetic studies have identified a number of statistically robust new genes and variants; however, these in themselves only show “association” with the phenotype. Thus, further analysis is needed to translate genetic findings into the knowledge of the molecular brain pathology of schizophrenia. This can be facilitated, for example, by testing if specific molecular and biological pathways are enriched among the associated genes.

**Fig. 2:**
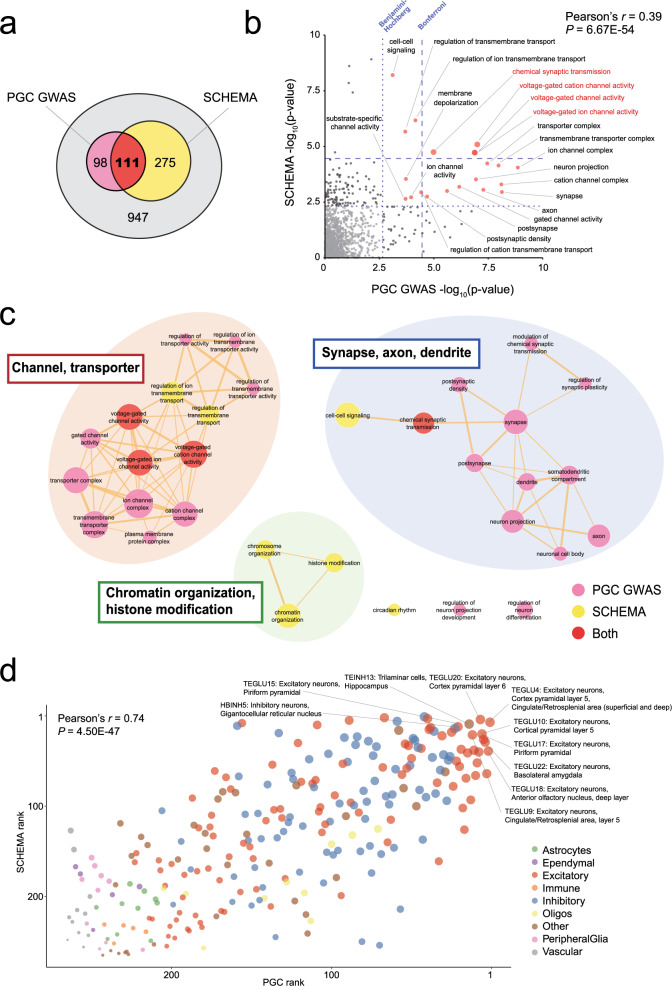
Functional convergence of the findings from studies of common and rare variants. **a** A Venn diagram showing the overlap of GO terms that showed enrichment (uncorrected P < 0.05) in the PGC GWAS and the SCHEMA exome sequencing study. **b** A plot of the enrichment of 1431 GO terms commonly analyzed in the PGC GWAS and the SCHEMA study. The *x*- and *y*-axes indicate −log_10_ uncorrected *P* values in the PGC GWAS and the SCHEMA study, respectively. The blue dotted lines indicate Bonferroni- or Benjamini-Hochberg-corrected significance thresholds. GO terms with Benjamini-Hochberg-corrected P < 0.05 in both studies are indicated by labeled red dots, of which four terms with Bonferroni-corrected *P* < 0.05 in both studies (voltage-gated cation channel activity, voltage-gated channel activity, voltage-gated ion channel activity, and chemical synaptic transmission) are labeled in red. The correlation between the two studies (Pearson’s *r* = 0.39. *P* = 6.67 × 10^−54^) is shown in the upper right. **c** Network visualization of the GO terms enriched in the PGC GWAS and the SCHEMA study. Significant GO terms after the Bonferroni correction in either or both studies are displayed. Nodes of significant GO terms are color-coded as follows: pink, the PGC GWAS; yellow, the SCHEMA study; red, both studies. The sizes of nodes are proportional to –log_10_ meta-analysis *P* values calculated by combining uncorrected *P* values in the PGC GWAS and the SCHEMA study using Fisher’s method. Nodes are connected when the similarity score ≥0.4, and the edge width is proportional to the similarity score. We observed three major clusters of GO terms, each related to channel or transporter activities; neuronal components (synapse, axon, and dendrite); chromatin or histone organization. **d** A plot of the results of cell type enrichment analyses in the PGC GWAS and the SCHEMA study. In both studies, the data of 265 cell types defined in the Zeisel et al. study [36] were used. The *x*- and *y*-axes indicate enrichment ranks in the PGC GWAS and the SCHEMA study, respectively. The top ten cell types for which the sum of the ranks in the PGC GWAS and the SCHEMA study is the smallest are labeled with the cell cluster ID, major cell type, and likely location. Each circle indicates each cell type, which is color-coded as indicated in the lower right. The sizes of the circles are proportional to the enrichment ranks. The correlation between the two studies (Pearson’s *r* = 0.74. *P* = 4.50 × 10^−47^) is shown in the upper left.

More specifically, gene ontology (GO) enrichment analyses were performed in both the PGC GWAS and the SCHEMA study by utilizing MAGMA [34] and DNENRICH [16] that appropriately control for confounding factors such as gene sizes and linkage disequilibrium. We, therefore, examined how the results of these two studies converge at the levels of molecular function. In the PGC GWAS and the SCHEMA study, the results of GO enrichment analysis for 7315 and 1491 terms are available, respectively. Of these, 1431 GO terms were commonly analyzed and 111 of them showed significant enrichment at uncorrected *P* < 0.05 in both of these two studies (Fig. 2a). The statistical significance (−log_10_ P value) for each term in the PGC GWAS and the SCHEMA study showed a highly significant correlation (Fig. 2b, Pearson’s correlation coefficient = 0.39, *P* = 6.67 × 10^−54^). This result indicates that there is a convergence of molecular and biological pathways implicated by common SNPs and rare deleterious variants. Specifically, four GO terms, all related to voltage-gated channels and synaptic transmissions, were significant after Bonferroni correction in both the PGC GWAS and the SCHEMA study (Fig. 2b). When the 32 GO terms with Bonferroni-corrected P < 0.05 in either dataset (respectively 25 and 11 terms in the PGC GWAS and the SCHEMA study, of which four are common as above) were visualized as networks by connecting them based on the similarity of the contained genes (Fig. 2c), we observed the formation of three clusters, each related to channel or transporter activities; neuronal components (synapse, axon, and dendrite); chromatin or histone organization. Though the cluster of chromatin or histone organization was only supported by SCHEMA, the former two clusters (“channel or transporter activities” and “neuronal components”) showed convergent enrichment in both studies. Taken together, these can be considered molecular pathways whose involvement in schizophrenia pathogenesis is supported by both common and rare variant studies.

In addition to molecular pathways, both the PGC GWAS and the SCHEMA study performed cell-type enrichment analysis utilizing data from single-cell RNA sequencing, which is rapidly developing in recent years. For this analysis, both studies employed the statistical method described by Skene et al. [35] and used the data of 265 cell types defined in a single-cell RNA sequencing study of the mouse nervous system by Zeisel et al. [36]. When we plotted the enrichment ranks for the 265 cell types in the PGC GWAS and the SCHEMA study, which are detailed in Supplementary Fig. 3 and Supplementary Table 10 of the corresponding papers, respectively, we again found that there is a highly significant correlation (Fig. 2d, Pearson’s correlation coefficient = 0.74, *P* = 4.50 × 10^−47^) (note that we used the enrichment ranks because exact statistics were not available in Supplementary Fig. 3 of the PGC GWAS). Overall, enrichment was particularly strong in excitatory neurons, followed by inhibitory neurons, and less pronounced in other cell types such as glial, vascular, and immune cells. Among the top ten highest-ranked cell types, eight were excitatory neurons, of which five, including the top two (“TEGLU4: Excitatory neurons, Cortex pyramidal layer 5, Cingulate/Retrosplenial area (superficial and deep)” [1st in PGC GWAS and 7th in SCHEMA) and “TEGLU20: Excitatory neurons, Cortex pyramidal layer 6” [7th in PGC GWAS and 4th in SCHEMA]), were annotated as deep layer excitatory neurons. These results represent another form of functional convergence of the findings from genetic studies of common and rare variants, and provide insight into the cell types likely playing a critical role in the molecular pathology of schizophrenia.

### Other types of variants potentially explain still-missing heritability

Although large-scale genetic studies and refinements in statistical methods have elucidated a substantial part of the genetic architecture of schizophrenia, there remains a large gap between the overall heritability reported in epidemiological studies (60–80%) [5–8] and that explained by common SNPs (24% in ref. [20]) or rare gene-disruptive rare SNVs, indels, and CNVs (<10% according to refs. [37, 38]). Provisional evidence suggests other types of variants that are not captured by GWAS or exome sequencing are likely to be involved, including rare non-coding variants [39] and tandem repeat variants [40] identified through whole genome sequencing. Also, there is preliminary evidence suggesting the role of postzygotic somatic variants [31, 41–44], while these are not transmitted and do not contribute to heritability. More detailed information on results from these pioneering but preliminary studies can be found in Supplementary Note. To more accurately estimate the contributions from these under-studied types of variants, further large-scale studies are mandatory.

## Transcriptomic and epigenomic pathology in schizophrenia

GO enrichment analysis of rare coding variants identified regulation of transcription and chromatin organization as one of the molecular pathways implicated in schizophrenia (Fig. 2c). Also, multiple single genes with large effect sizes, such as *SP4* encoding the transcription factor Sp4 and *SETD1A* encoding a histone methyltransferase, are core components of transcriptional and epigenetic regulation. In line with these findings, studies of transcriptomic and epigenomic pathology of schizophrenia using patient-derived tissues have been conducted at scale.

One of the largest studies of transcriptomic brain pathology in schizophrenia was conducted by the PsychENCODE consortium [45]. In this study, gene- and transcript isoform-level differential expression was comprehensively analyzed by performing RNA-sequencing (RNA-seq) of bulk postmortem cerebral cortex tissues from 559 schizophrenia cases and 936 control individuals, together with 51 ASD and 222 bipolar disorder brains. They identified that the expression of 4821 genes and 3803 isoforms significantly differed between schizophrenia and controls (FDR < 0.05). Genes related to “inflammatory response” and “receptor activity” were enriched in the significantly upregulated and downregulated genes/isoforms, respectively. The enrichment of the genes related to receptor activities is consistent with the results summarized in the previous section. Schizophrenia heritability was enriched among genes and transcripts dysregulated in schizophrenia brains, especially in down-regulated transcript isoforms.

Regarding epigenomic brain pathology, a recent large-scale study analyzed two major histone modifications, histone 3 lysine 27 acetylations (H3K27ac) and histone 3 lysine 4 trimethylations (H3K4me3) [46], in postmortem prefrontal cortical samples (sorted neurons or bulk tissues) from 303 schizophrenia cases and 388 controls along with 48 bipolar disorder brains by chromatin immunoprecipitation sequencing (ChIP-seq) [47]. In the analysis of differential H3K4me3/H3K27ac peaks, 6219 differential H3K27ac peaks (FDR < 0.05) between schizophrenia and controls were identified though there were no differential H3K4me3 peaks. Of these, schizophrenia heritability based on GWAS [20] was enriched in the H3K27ac peaks hyper-acetylated in schizophrenia. Subsequently, this study mapped cis-regulatory domains (CRDs), which often overlap with topologically associating domains defined by the analysis of 3D chromosomal conformations but constitute smaller regulatory units of 10^4^–10^6 ^bp [48, 49], in the brain using the information of inter-individual correlations between histone peaks. In an analysis integrating information on CRDs and differential H3K27ac peaks, it was shown that schizophrenia heritability is strongly enriched at differential H3K27ac peaks in CRDs hyper-acetylated in schizophrenia, suggesting that dysregulated H3K27ac peaks within dysregulated CRDs particularly are associated with the genetic schizophrenia risk.

Besides histone modifications, DNA methylation is another major epigenetic modifier with regulatory functions. Several studies have explored genome-wide DNA methylation status in postmortem schizophrenia brains. Among them, a study of microarray-based analysis of DNA methylation at ~450,000 loci in postmortem dorsolateral PFC (DLPFC) tissues from 526 individuals was reported [50]. In this study, a total of 2104 sites differentially methylated between quality-controlled 108 schizophrenia cases and 136 controls (Bonferroni-corrected *P* < 0.05), of which 97.1% were hypomethylated in schizophrenia, were identified. A GO enrichment analysis of genes in or near the differentially methylated sites showed an overrepresentation of genes related to embryo development, cell fate commitment, and nervous system differentiation. Also, modest enrichment of schizophrenia-associated loci among the differentially methylated sites (1.9% among differentially methylated sites vs. 1.3% among others, *P* = 0.004, Chi-square test) was observed. On the other hand, in a study of postmortem brain samples overlapping with the above-described microarray-based study (70 and 95 schizophrenia DLPFC and hippocampus, and 77 and 102 control DLPFC and hippocampus) using whole-genome bisulfite sequencing, a technique that can detect DNA methylation at the single base resolution, much smaller numbers of differentially methylated sites, none in DLPFC and 70 in the hippocampus, were identified despite less stringent multiple testing corrections (FDR < 0.05) [51]. This discrepancy could be explained by the difference in sample sizes as well as the sensitivity to detect differentially methylated sites and the number of hypotheses tested, as a larger number of sites are covered by whole-genome bisulfite sequencing.

In addition to the studies using postmortem brain tissues, there are large-scale studies of peripheral samples aiming to identify disease biomarkers. In a study by Aberg et al. [52], analyzing genome-wide DNA methylation profiles in blood samples from 759 schizophrenia cases and 738 controls by methyl-CpG–binding domain protein-enriched genome sequencing, 25 and 139 sites associated with the diagnosis at Bonferroni-corrected *P* < 0.05 and FDR < 0.01, respectively, were identified. The most significant association was observed in the region involving *FAM63B*, a part of networks regulated by microRNAs associated with neuronal differentiation and dopaminergic gene expression. This association was replicated in an independent cohort of >1000 individuals. The observed effect sizes for three associated methylation sites at *FAM63B* were moderate (Cohen’s *d* = 0.42–0.45). In a recent meta-analysis of blood DNA methylation profiles from 4483 participants from seven cohorts, including 1681 schizophrenia cases and 1583 controls, by Hannon et al. [53], 1013 differentially methylated loci with methylome-wide significance (P < 9 × 10^−8^), which were annotated to 692 genes, were identified. Among 158 schizophrenia-associated loci identified by GWAS [10], overall differential DNA methylation was observed at 21 loci after correcting for multiple testing, supporting co-localization of signals from GWAS and epigenome-wide association study. On the other hand, an integrative analysis of DNA methylation and genetic variants exploring the causal relationships was not performed in their study. Further studies of the interaction between genetic and epigenetic factors that are expected to provide additional insights into the molecular mechanisms underlying co-localization are warranted.

Overall, while the significant overlap between differentially expressed or modified genes and the genetic risk of schizophrenia has been reported in some studies, transcriptomic or epigenomic alterations of single genes that can biologically define the general population of schizophrenia or serve as a high-sensitivity and specificity biomarker have not been discovered, or perhaps there is no such universal molecular marker. Therefore, further studies are necessary to identify conclusive transcriptomic and epigenomic pathology in schizophrenia.

## Studies of etiologically valid mouse and cellular models of schizophrenia

As summarized in Table 1 and Fig. 1, recent large-scale genetic studies have identified specific genes, SNVs/indels, and CNVs that confer a substantial risk of schizophrenia. Based on this, rodents or cells carrying the alleles equivalent to the above-described risk variants identified in humans have been generated and analyzed. In this section, we overview studies of such etiologically valid, i.e., having the same causal conditions as in human patients, models of schizophrenia. (Tables 2 and 3), which have provided various insights into the connection between genetic pathology and pathological changes at the levels of molecules (e.g., transcripts and proteins), cells, neural circuits, whole tissues, or individuals’ behaviors.

**Table 2:** Etiologically valid mouse models of schizophrenia.

Risk variant in human	Type of modification	Gene editing strategy	Common mutant allele name	Zygosity	Background	Ref. number	First author	Year	Key molecular phenotypes	Key cellular/circuity phenotypes	Other findings	Behavioral phenotypes				
												Sociality	Cognitive function	ASR	PPI	Other behaviors
22q11.2 deletion	1.3 Mb deletion of chr16	Gene-targeting	Df(16)A+/-	Heterozygous	C57BL/6J	54	Stark KL.	2008	• Altered miRNA expression explained by *Dgcr8* haploinsufficiency • Altered expression of "oxidative phosphorylation" and "ATP-synthesis-coupled electron transport" genes in PFC and "Transmission of nerve impulse" and "Synaptic transmission" genes in the hippocampus	• Decreased width of mushroom spines and reduced dendritic complexity in heterozygous knockout mice of *Dgcr8* within 22q11.2			Impaired	Normal	Low	Hyperactive, anxiety in male mice
				Heterozygous	C57BL/6J	55	Mukai J.	2008		• Decreased density of dendritic spines, glutamatergic synapses, and dendritic complexity • Lower miniature EPSC frequency of pyramidal neuron	• Rescue of the morphological alteration by restoration of *Zdhhc8* in 22q11.2					
				Heterozygous	C57BL/6J	56	Sigurdsson T.	2010		• Reduced hippocampal-prefrontal synchrony						
				Heterozygous	C57BL/6J	57	Xu B.	2013	• Lower expression of miR-185 within 22q11.2 • Higher expression of *Mirta22* (*Emc10*), a target of miR-185	• Decreased spine density and dendritic complexity, rescued by either upregulation of miR-185 or downregulation of Mirta22						
				Heterozygous	C57BL/6J	58	Mukai J.	2015		• Disrupted axonal growth, branching, and synaptic transmission (smaller fEPSC in the frontal cortex)	• Altered morphology of neurons and impaired prefrontal-hippocampus synchrony in *Zdhhc8*^+/-^ mice					
				Heterozygous	C57BL/6J	59	Hamm JP.	2017		• Neuronal activity with the single-cell resolution was not altered, but neuronal coactivity patterns (ensembles) were altered						
	around 1 Mb deletion of chr16	Gene-targeting	Df(16)1/+	Heterozygous	C57BL/6J	60	Chun S.	2014		• Disruption of synaptic transmission at thalamocortical glutamatergic projections in the auditory cortex caused by aberrant elevation of *Drd2* in the thalamus • Rescue effect of an antipsychotic, haloperidol and clozapine, on the abnormal thalamocortical projection	• Abnormal thalamocortical sensitivity to haloperidol • Deficits of PPI in Dgcr8+/- mice • Rescue of the deficiency of PPI in *Dgcr8*+/- mice by haloperidol					
	3.0 Mb deletion of chr16	CRISPR/Cas9	Del(3.0Mb)/+	Heterozygous	C57BL/6N	61	Saito R.	2020	• Decreased expression of miR-185-5p	• Attenuation of visual-evoked potential of the primary visual cortex (V1) and the frontal cortex		Normal	Impaired	Increased	Low	Hypoactivity
16p11.2 deletion	0.39 Mb deletion of chr7	Cre-mediated recombination	Del(7Slx1b-Sept1)4Aam	Heterozygous	C57BL/6N x 129Sv	62	Horev G.	2011			• Generation of the mice • Enlargement of multiple brain structures	Normal				Altered diurnal rhythms
				Heterozygous	C57BL/6 x 129Sv	63	Blumenthal I.	2014	• Altered expressions "regulation of transcription" and "regulation of actin cytoskeleton organization" genes in the cortex							
				Heterozygous	C57BL/6 x 129Sv	64	Brunner D.	2015			• Growth deficits	Normal	Normal	Normal	Normal	Normal USVs
				Heterozygous	B6129SF1/J	65	Yin X.	2021		• Delayed spine pruning • Abnormally high ensemble activity of layer II-III excitatory neurons in the motor cortex during the initial phase of learning						Delayed motor learning
	0.44 Mb deletion of chr7	Cre-mediated recombination	Del(7Coro1a-Spn)1Dolm	Heterozygous	C57BL/6N	66	Portmann T.	2014	• Altered dopaminergic signaling in neonatal brain	• Increased number of striatal medium spiny neurons with dopamine D2 receptor (Drd2) and fewer Drd1+ neurons in deep layers of cortex • Abnormal basal ganglia circuitry function	• Generation of the mice • Growth deficits • Anatomical abnormalities in juvenile mice	Normal	Impaired	Decreased		Hyperactivity
				Heterozygous	C57BL/6N	67	Yang M.	2015			• Growth deficits	Reduced anogenital sniffing		Decreased (deafness)	Low (deafness)	Fewer USVs, reduced pain sensitivity
	0.46 Mb deletion of chr7	Cre-mediated recombination	Del(7Sult1a1-Spn)6Yah	Heterozygous	C57BL/6N	68	Arbogast T.	2016			• Underweight despite human carriers showing overweight	Normal	Impaired			Increased repetitive behaviors, increased center time in open field
16p11.2 duplication	0.39 Mb duplication of chr7	Cre-mediated recombination	Dp(7Slx1b-Sept1)5Aam	Heterozygous	C57BL/6N x 129Sv	62	Horev G.	2011			• Generation of the mice	Normal				Altered diurnal rhythms, increased grooming and resting
					C57BL/6 x 129Sv	63	Blumenthal I.	2014	• Altered expressions of "regulation of transcription" and "response to protein stimulus" genes in the cortex							
					C57BL/6 x 129S7	69	Blizinsky KD.	2016	*•* Identification of *Mapk3* within 16p11.2 as a central hub in schizophrenia-associated CNV protein-protein interaction network	• Increased dendritic arborization	• Reversal of dendritic phenotypes by MEK inhibitor					
					C57BL/6	70	Rein B.	2021	• Upregulation of enzyme modulator genes and downregulation of transcription factor genes, including GABAergic synapse regulator *Npas4* in PFC	• Deficient GABAergic synaptic transmission and elevated neuronal excitability in the prefrontal cortex	• Rescue of synaptic and behavioral phenotypes by restoration of *Npas4* in PFC	Low	Impaired	Normal	Normal	Increased self-grooming, hypoactivity
	0.46 Mb duplication of chr7	Cre-mediated recombination	Dp(7Sult1a1-Spn)6Yah	Heterozygous	C57BL/6N	68	Arbogast T.	2016		• Smaller LTP in CA1	• Overweight despite human carriers showing underweight	Normal	improved			Hypoactivity, decreased center time in open field test
3q29 deletion	1.22 Mb deletion of chr16	Cre-mediated recombination		Heterozygous	C57BL/6J	71	BaBa M.	2019		• Neuronal hyperactivation and reduction of parvalbumin-positive cells in the cortex	• Growth deficits, lower brain weight	Low	Impaired	Increased	Low	Hyperactivity, increased self-grooming
	1.26 Mb deletion of chr16	CRISPR/Cas9		Heterozygous	C57BL/6N	72	Rutkowski TP.	2021			• Growth deficits • Partial but not complete reproduction of the phenotypes in the deletion model in mice with haploinsufficiency of *Dlg1* within 3q29	Low	Impaired in male Normal in female	Normal in male Increased in female	Normal	
15q11.2-13.1 duplication	6.3 Mb duplication of chr7	Cre-mediated recombination	Dp(7Herc2-Mkrn3)1Taku	Heterozygous	C57BL/6J or 129SvEv	73	Nakatani J.	2009		• Altered 5-HT2c receptor signaling		Low	Longer freezing in cued condition of FC test	Increased	Normal	Fewer USV, inflexibility, increased anxiety, depressive behavior
2p16.3 (*NRXN1*) deletion	Deletion of exon1	Gene-targeting	Nrxn1^tm1Sud^	Homozygous	SV129xC57BL/6	74	Geppert M.	1995			• Generation of Nrxn1α knockout mice					
				Homozygous	SV129xC57BL/6	75	Etherton MR.	2009		• Reduced spontaneous excitatory synaptic transmission in CA1 pyramidal neurons • Decreased evoked excitatory synaptic strength in CA1		Normal	Normal	Increased	Low	Increased self-grooming, impaired nest-building behaviors
				Homozygous	C57BL/6J	76	Grayton HM.	2013			• No significant behavioral changes in heterozygous KO mice	Aggressive behavior Increased social interaction	Normal			Impairment of locomotor activity in females, anxiety in males, impairment of nest-building behavior
				Heterozygous	SV129xC57BL/6	77	Dachtler J.	2015				Impaired discrimination	Impaired in female	Normal	Normal	
				Homozygous/ heterozygous	C57BL/6	78	Davatolhagh MF.	2021		• Decreased synaptic strength specifically onto indirect pathway spiny projection neurons in dorsal PFC-dorsomedial striatum (DMS) circuit (heterozygous and homozygous)	• Altered postsynaptic NMDAR function at parafascicular-DMS synapses (homozygous only)					
*GRIN2A* LOF/PTV	Disruption of the exon 10 (knockout)	Gene-targeting	Grin2a^tm1Mim^	Homozygous	C57BL/6 x CBA	79	Sakimura K.	1995		• Reduced channel current of NMDA receptor and hippocampal LTP in CA1 pyramidal cells	• Generation of the mice		Impaired			
				Heterozygous	C57BL/6	80	Takeuchi T.	2001						Normal	Normal	Homozygous knockout mice showed increased startle responses
				Heterozygous	C57BL/6J	81	Marquardt K.	2014					Normal			Homozygous knockout mice showed cognitive dysfunction in set-shifting task
	Disruption of the exon 10 (knockout)	Gene-targeting	Grin2^atm1Nak^	Homozygous	129/SvJ	82	Kadotani H.	1996		• Reduced amplitude of the slow component of EPSC in mossy fiber-granule cells in cerebellar slices	• Generation of the mice					Normal motor coordination
				Heterozygous	C57BL/6	83	Spooren W.	2004			• Impaired PPI by Inhibition of both GRNI2A/B			Normal	Normal	Homozygous knockout mice did not show any significance in PPI
*GRIA3* LOF/PTV	Disruption of exon 12	Gene-targeting	Gria3^tm1Zpj^	Hemizygous	129 x C57/BL6	84	Meng Y.	2003		• Normal basal synaptic transmission • Normal presynaptic function and LTD but enhanced LTP	• Generation of the mice					No apparent behavioral deficits (data not shown)
				Hemizygous	C57BL/6J	85	Peng SX.	2022		• Decreased excitation of mPFC neurons	• Improvement of aggression by restoration of *Gria3* in mPFC	Aggressive behavior				Reduced anxiety in the elevated plus maze
	Disruption of exon 12	Gene-targeting	Gria3^tm2Rlh^	Hemizygous	C57BL/6J	86	Adamczyk A.	2012		• Increased dopamine concentrations in striatum and reduced serotonin turnover in the olfactory bulb		Aggressive behavior	Normal			Impaired motor function, normal anxiety
	Disruption of exon 12	Gene-targeting	Gria3^tm1Dgen^	Hemizygous	C57BL/6	87	Renner MC.	2017		• Impaired cAMP-driven postsynaptic potentiation of CA1 neurons						
*SETD1A* LOF/PTV	Premature stop codon cassette in intron 3	Gene-targeting	Setd1a^tm1a(EUCOMM)Wtsi^	Heterozygous	C57BL/6J	88	Mukai J.	2019	• Enrichment of high-confidence target genes of Setd1a for synaptic and established schizophrenia/NDD-associated genes	• Decreased mushroom spine density and axonal branching but not dendritic complexity in PFC neurons • Altered neuronal short-term plasticity and excitability in PFC	• Significant overlap between Setd1a and Mef2 targets at enhancers	Normal	Impaired			
				Heterozygous	C57BL/6J	89	Hamm JP	2020		• Aberrant cortical cell-cell ensembles in V1 during ongoing and visual evoked activities • Altered cortical oscillations						
				Heterozygous	C57BL/6J	90	Bosworth ML.	2021						Increased	Low	Increased time spent in inner zone in open field test
	2 bp deletion in exon 7	CRISPR/Cas9		Heterozygous	C57BL/6N	91	Nagahama K.	2020	• Enrichment of DEGs in mPFC for neural/synaptic/axonal genes and schizophrenia/NDD-associated genes	• Attenuated excitatory synaptic function and altered structure such as smaller number of spines in L2/3 pyramidal neurons of the medial PFC		Low	Impaired	Normal	Low	Hypoactivity
	8 bp deletion in exon 15	CRISPR/Cas9		Heterozygous	C57BL/6N	92	Chen R.	2022	*•*Larger numbers of DEGs in *Foxp2*(+) deep layer cortical neurons and striatal D1- or D2-type medium spiny neurons • Enrichment of synaptic genes in PFC excitatory neuron DEGs	• Impaired exocytosis functions • Decreased spine density and dendritic complexity in PFC and striatal neurons	• Reduced H3K4me3 in *Foxp2* (+) neurons	Normal	Normal	Increased	Low	Lower sucrose preference

*ASR* acoustic startle response, *DA* dopamine, *DEG* differentially expressed gene, *EPSC* excitatory postsynaptic current, *FC* fear conditioning, *fEPSC* field excitatory postsynaptic current, *iPS* induced pluripotent stem cell, *LOF* loss-of-function, *LTD* long term depression, *LTP* long term potentiation, *mPFC* medial PFC, *NDD* neurodevelopmental disorders, *NPC* neural progenitor cell, *PFC* prefrontal cortex, *PPI* prepulse inhibition, *PTV* protein-truncating variant, *USV* ultrasonic vocalization.

**Table 3:** Etiologically valid cellular models of schizophrenia.

Risk variant in human	Model type	Differentiation	Zygosity	N (Case)	N (Control)	Ref. number	First author	Year	Molecular phenotypes	Cellular/circuity phenotypes	Other findings
22q11.2 deletion	Patient-derived	Neurosphere	Heterozygous	2	4	94	Toyoshima M.	2016	• Downregulation of miR-17/92 and miR-106a/b clusters	• Reduction of neurosphere size, neural differentiation, neurite outgrowth, cellular migration, and neurogenic-to-gliogenic competence ratio	
	Patient-derived	NPC, 2D-neurons	Heterozygous	8	7	95	Lin M.	2016	• Upregulation of genes related to apoptosis/programmed cell death and downregulation of synaptic, cell cycle, and microtubule organization genes	• Lower NPC-proliferation rates	
	Patient-derived	Cerebral cortical organoids	Heterozygous	15	15	96	Khan TA.	2020	• Altered expressions of neuronal excitability-related genes • Enrichment of schizophrenia heritability in DEGs of mature organoids	• Deficits of neuronal excitability and calcium signaling	• Recapitulation of calcium and membrane potential alterations in *DGCR8*^+/-^ neurons • Rescue by *DGCR8* restoration or an antipsychotic, raclopride
	Patient-derived	iPS, NPC, 2D-excitatory neurons	Heterozygous	20	29	97	Nehme R.	2022	• Enrichment of schizophrenia genetic risk and synaptic genes in upregulated DEGs of NPCs and neurons	• Lower spiking rate of neurons in multi-electrode array analysis	
16p11.2 deletion/duplication	Patient-derived	2D-cortical neurons	Heterozygous	3 deletion 3 duplication	4	98	Deshpande A.	2017		• Increased and decreased soma area/dendritic length in 16p11.2 deletion and duplication, respectively • Reduction of synaptic density in both deletion and duplication	
	Patient-derived	Cortical organoids	Heterozygous	6 deletion 8 duplication	4	99	Kostic M.	2021	• A larger number of DEGs in 16p11.2 deletion than in duplication • Downregulation of neuron projection morphology-related genes and upregulation of positive chemotaxis genes in the deletion lines • Perturbation of genes regulated by RBFOX1 in the deletion lines	• Similar cell composition across organoids derived from 16p11.2 CNV carriers (deletion or duplication) and controls as revealed by scRNA-seq	
	Patient-derived	Cortical organoids	Heterozygous	3 deletion 3 duplication	3	100	Urresti J.	2021	• Enrichment of “ligand-gated ion channel activity” genes in DEGs of 16p11.2 deletion • No enrichment of GO terms in DEGs of 16p11.2 duplication	• Accelerated neural maturation in 16p11.2 deletion than in duplication • Increased synaptic puncta in 16p11.2 deletion • Neuronal migration deficits in both 16p11.2 deletion and duplication	• Rescue of migration deficits by inhibition of RhoA activity
	CRISPR/Cas9	2D-dopaminergic neurons	Heterozygous	3 deletion 3 duplication	3	101	Sundberg M.	2021	• Increased synaptic marker expression in 16p11.2 deletion lines • Reduced synaptic marker expression in 16p11.2 duplication lines	• Increased soma size in 16p11.2 deletion • Deficits in neuronal differentiation in 16p11.2 deletion	• Hyperactive DA neuronal networks and increased bursting in 16p11.2 deletion
	CRISPR/Cas9	2D-neurons and cerebral organoids	Heterozygous	7 deletion 6 duplication (in the 2D-neuron assay)	6 clones (in the 2D-neuron assay)	102	Tai DJC.	2022	• Only nine DEGs in 16p11.2 deleted 2D-neurons while 95 DEGs in duplicated neurons • Enrichment of energy metabolism-related genes in DEGs of 16p11.2 duplicated 2D-neurons	• Shortened neurites with reduced branchpoints in both 16p11.2 deleted and duplicated 2D-neurons • Reduced activity, synchrony, and oscillation in 2D-neurons	• Altered proportion of the cell components of cerebral organoids in 16p11.2 deletion (i.e., decreased excitatory neurons and increased inhibitory GABAergic neuron) (2 clones per genotype in the cerebral organoid assays)
2p16.3 (*NRXN1*) deletion	Deletion of exon 19 shared by all neurexin-1 isoforms or insertion of premature stop codon just before the last exon by Cre-loxP/FLP-FRT in human ES cells	2D-excitatory cortical neurons	Heterozygous	1 (exon 19 deletion) 2 (premature stop codon)	1 (exon 19 deletion) 2 (premature stop codon)	103	Pak C.	2015		• Normal neuronal morphology • Decreased spontaneous mEPSC frequency • Iimpaired neurotransmitter release	
	Patient-derived	2D-neurons	Heterozygous	2 (5’-*NRXN1a*^+/-^) 2 (*NRXN1a/b*^+/-^)	4 (including a family member of *NRXN1α/β*^+/-^ carrier)	104	Flaherty E.	2019	• Enrichment of genes related to transcriptional/epigenetic regulation and schizophrenia GWAS-associated genes in DEGs of patient-derived NPC and neurons	• Reduced spontaneous neuronal activity in multi-electrode array analysis	• Comprehensive cataloging of a total of 123 high-confidence in-frame human *NRXN1α* isoforms
	Patient-derived	2D-neurons resembling cortical layer 2/3 pyramidal neurons	Heterozygous	3 carrier-derived	3 non-carrier-derived	105	Pak C.	2021	• No consistent differences between carrier- and non-carrier-derived neurons in principle component analyses of the data of bulk-RNA-seq • Upregulation of intracellular NRXN1-binding protein CASK in patient-derived iPS-neurons	• No change in dendritic and synaptic morphologies • Decrease in the frequency of spontaneous excitatory synaptic events, in evoked excitatory synaptic transmission, and in paired-pulse depression	
*SETD1A* LOF/PTV	CRISPR/Cas9 (heterozygous frameshift deletion in exon 7)	2D-neurons	Heterozygous	2 clones	1 clone	106	Wang S.	2022	• Perturbation in gene sets associated with glutamatergic synaptic function • Increased cAMP signaling	• Increase of dendritic length and arborization, network burst activity, and synaptic integration	

*DEG* differentially expressed gene, *GWAS* genome-wide association study, *iPS* induced pluripotent stem cell, *NPC* neural progenitor cells, *scRNA-seq* single-cell RNA sequencing.

### Mouse models

We systematically surveyed studies of mice with mutant alleles orthologous to the variants listed in Table 1 with an observed OR greater than ten. We found that there are studies on the following variants: 22q11.2 deletion, 16p11.2 deletion/duplication, 3q29 deletion, 15q11.2–13.1 duplication, 2p16.3 (*NRXN1*) deletion, *GRIN2A* LOF/PTV, *GRIA3* LOF/PTV, and *SETD1A* LOF/PTV (Table 2) [54–92]. While there are studies of mice with mutations in other genes, such as *RB1CC1* (also known as *FIP200*), the introduced alleles were not equivalent to ones in human patients, and/or the mice were not analyzed in the context of neuroscience, and thereby not highlighted in this review.

Regarding the molecular phenotypes mainly analyzed by transcriptomic profiling, commonly dysregulated pathways include neural transmission and regulation of transcription [54, 63, 70, 88, 91, 92], in agreement with the findings from human genetics and genomics studies. Also, analyses utilizing results of large-scale human genetics studies have reported enrichment of genetic risk for schizophrenia in genes differentially expressed in the models or molecular targets of the genetically modified genes [88, 91]. Meanwhile, mutant mice originally created not as a schizophrenia model but to elucidate gene function in the central nervous system, such as knockout mice for *GRIN2A* or *GRIA3* encoding a glutamate receptor subunit, have not been subjected to omics analysis. Molecular profiling of these etiologically valid models may provide further convergent insights into the brain pathology of schizophrenia.

Morphological analysis of neuronal cells in these models has reported reduced axonal and dendritic complexity and abnormal spine morphology in *Setd1a* heterozygous knockout mice and several mouse models with CNVs [54, 55, 57, 58, 88, 91]. Common electrophysiological phenotypes include altered synaptic transmissions, such as diminished excitability indicated by reduced excitatory postsynaptic currents [55, 58, 60, 75, 79, 82, 85, 88, 91] or deficits in long-term potentiation [68, 79], though some mice showed increased excitability or altered activities of other neuronal subtypes such as GABAergic neurons [70, 84].

Behavioral alterations common in these mice include deficits in sociality, cognitive performance, and prepulse inhibition [54, 61, 66–68, 70–73, 75–77, 79, 88, 90–92]. These phenotypes are generally consistent with those in human schizophrenia patients [93], though there would be biases that these phenotypes are more likely to be investigated and reported. Also, in some cases, there were inconsistent results even among models with mutations in the same gene. This could be explained by differences in the method of introduction of the mutations (e.g., CRISPR/Cas9 or gene targeting), genetic backgrounds (e.g., C57BL/6J and C57BL/6N), and other factors. In addition, the acquisition of more definitive results will be facilitated by strict standardization of analysis protocols and ensuring a sufficient sample size, as have been done in human genetics studies.

### Cellular models

Recent technological advances have enabled the reproduction of pathological conditions in vitro by creating patient-derived or mutation-carrying induced pluripotent stem (iPS) cells and then differentiating them into central nervous system cells or miniature brains. Studies of etiologically valid cellular models of schizophrenia produced with this technology, including those with 22q11.2 deletion, 16p11.2 deletion/duplication, *SETD1A* LOF/PTV, and *NRXN* LOF/PTV, have been conducted and reported [94–106] (Table 3).

In line with the findings in etiologically valid mouse models, molecular profiling of these cellular models generally supports dysregulation of genes related to neural transmission, especially synaptic genes [95–97, 100, 101, 106], transcriptional regulators including microRNAs [94, 104], and schizophrenia-associated genes discovered by human genetics studies [96, 97, 104]. Also, morphological alteration of soma and dendrite were common except for iPS cell-derived neurons with *NRXN1* deletion [94, 98, 101, 102, 106]. Abnormal neural activities are identified in multiple patient-derived or genetically engineered iPS cell-derived neurons, however, the directions of the abnormalities are sometimes inconsistent across models manipulating different genes [97, 101–106]. Though it may be due to artifacts depending on the differences in the experimental designs, another possibility is that imbalanced excitatory/inhibitory activities themselves [107], regardless of the direction of abnormality, are important in schizophrenia pathology. It is also worth noting that multiple studies have investigated interventions to improve abnormal phenotypes observed in cellular models [96, 100]. Overall, the iPS cell technology is a powerful tool to analyze the molecular pathology of schizophrenia using human samples, and further studies are warranted.

### Considerations on the model validity

In the previous sections, we have defined etiologically valid models using the criteria that the modified gene showed a significant association with schizophrenia after stringent multiple testing correction and that the ORs observed in studies that found the association was greater than ten. However, we would like to explicitly state that there is still uncertainty regarding the validity of these models.

First, it should be noted that in general, there are wide ranges of confidence intervals for ORs for rare risk variants (Fig. 1 and Table 1). Indeed, another large-scale study (20,403 cases and 26,628 controls) analyzing the association of schizophrenia and CNVs implicated in neurodevelopmental disorders reported no statistically significant associations of 16p11.2 deletion and 15q11.2–13.1 duplication with schizophrenia and association of 2p16.3 (*NRXN1*) deletion with OR smaller than ten [30, 32]. Therefore, there is the possibility that ORs are overestimated in the existing data. Second, as mentioned in the section on genetic pathology, many of the genes and variants highlighted here, especially almost all CNVs, are associated not only with schizophrenia but also with other neurodevelopmental disorders. Given this, the mice and cells harboring such mutations should not be considered as specifically modeling schizophrenia. Third, particularly in the case of CNV-based animal models, evaluation of the model validity and the interpretation of the phenotypes require caution because CNVs usually contain multiple genes and non-coding regions whose structure is sometimes not well conserved between rodents and humans. Lastly, we would like to emphasize that a number of valuable findings interpreted as being relevant to the molecular pathology of schizophrenia have also been obtained from analyses of models in which genes not strongly supported by currently available evidence from human genetics studies were manipulated. For example, given the mechanisms of action of known antipsychotics, it is quite obvious that dysregulation of the monoaminergic system is involved in the pathophysiology of schizophrenia [108, 109], and therefore various genes in this pathway have been intensively investigated. Although these include genes that do not have strong genetic support, unlike *DRD2* identified in GWAS [9, 20] and others, they form a foundation not only for the study of schizophrenia patients but also for the study of animal and cellular models with genetic etiological validity [60, 110–114]. Besides, as evidenced by the fact that heritability is better explained by considering numerous SNPs, not only those in genome-wide significant loci, it is clear that there are true schizophrenia risk genes among those that did not reach the stringent significance threshold with the current sample sizes. This indicates that further identification of robust risk genes in larger studies will certainly increase the value of existing research using animal and cellular models with modifications of such genes. Meanwhile, it is also true that there are mice that have been interpreted as models of schizophrenia based on their face validity, despite the lack of etiological validity in light of currently available knowledge. Moreover, sometimes models are interpreted as meeting face validity based on phenotypes in homozygous mutants, even though heterozygous variants are associated with schizophrenia in humans. Therefore, caution should be exercised in discussing the validity of a model solely on the strength of its face validity, regardless of the robustness of the association between the manipulated gene and schizophrenia risk.

## Current achievements, limitations, and new directions for future research

Based on the above-described existing knowledge of the genetic and molecular pathology of schizophrenia as well as the emerging insights into their links to other scales of pathologies from studies of etiologically valid models, we summarize the current achievements, limitations, and new directions for future research as follows.

Regarding the genetic pathology, significant advances, such as the elucidation of the highly polygenic nature of schizophrenia, the explanation of more than 20% of disease liability by measurable genetic variants, and the identification of specific genes and CNVs associated with schizophrenia with large effect sizes, have been achieved. While a substantial part of the heritability is still unexplained and the number of disease-responsible genes identified so far is not as large as in ASD, where similarly large studies have been conducted, it is certainly expected that by simply increasing the sample size and investigating under-studied types of variants, such as rare non-coding mutations, we can better explain the genetic liability to schizophrenia and identify additional responsible genes. Indeed, a more recent target sequencing study of candidate schizophrenia-associated genes in 11,580 cases and 10,555 controls, followed by a meta-analysis with the SCHEMA dataset identified two novel exome-wide significant genes, *AKAP11* and *SRRM2* [115], confirming the importance of increasing sample sizes. In addition to the promotion of basic genetic research, the application of genetic information to clinical psychiatry is a subject of active discussion. As an example, there are attempts to utilize polygenic risk scores (PRS) based on the profiles of common SNPs to predict clinical courses [116, 117], though further studies are needed. Also, the identification of patients with Mendelian genetic disease among patients clinically diagnosed with schizophrenia based on operationalized criteria and the optimization of their treatment based on genetic diagnosis is expected to be implemented in the near future.

Compared to the knowledge of genetic pathology, our understanding of the molecular pathology of schizophrenia, such as transcriptomic and epigenomic alterations, is insufficient and no convincing single molecular markers that can biologically define the schizophrenic brain have been identified. Nevertheless, collectively interpreting the results of large-scale omics analyses of human postmortem brains and studies of models with high etiological validity, one might be able to argue that small transcriptional and/or epigenetic alterations of many schizophrenia-associated genes and genes involved in neuronal processes such as the formation and regulation of synapses would be the underlying molecular brain pathology of schizophrenia. To obtain a clearer picture, the following directions would be considered.

First, as summarized in Tables 2 and 3, multiple etiologically valid models of schizophrenia have been generated and analyzed. One of the next important steps will be to elucidate the alterations that are commonly observed across them, and studies seeking this goal should be facilitated by investigating two or more models in the controlled same experimental settings. This is because the results of studies of disease models are often confounded by subtle differences in experimental design and conditions, such as apparatus, experimenter, mouse strain and genetic background, and others. However, to our knowledge, there have been no publications reporting the results of the analysis of multiple schizophrenia models with high etiological validity listed in Tables 2 and 3 under the same conditions. By conducting such studies of multiple models, which have already been done for ASD [118–121], it is expected that we can obtain convergent insight into the pathological changes in schizophrenia.

Second, while it must be recognized that collecting human postmortem brains on a large scale requires a great endeavor, there is an open question of whether the sample sizes in studies to date are sufficient. Evaluating the inter-study reproducibility, which may help answer this question, it is true that the result of the analysis of genes differentially expressed between schizophrenia cases and controls in the aforementioned PsychEncode study [45] is well correlated with that of a preceding study by the CommonMind Consortium (CMC) [122] (correlation coefficient between the two studies for 687 genes with FDR < 0.05 in the CMC study = 0.799). On the other hand, of the 23 genes that showed statistical significance after Bonferroni correction in the CMC study (uncorrected *P* < 3.04 × 10^−6^, 0.05 divided by the number of genes analyzed, 16,423), only nine genes surpassed the same significance threshold in the PsychENCODE study. This number is much more than random expectation; however, this contrasts with the observation in GWAS that 116 out of the 128 loci genome-wide significantly (*P* < 5 × 10^−8^) associated with schizophrenia in the previous PGC GWAS in 2014 were replicated with the genome-wide significant association in the same local regions in 2022 GWAS (and supporting evidence was obtained for 11 of the remaining 12 loci in an extended analysis). Given these, it is warranted to further increase the sample sizes in postmortem brain studies of schizophrenia in order to obtain robust and reproducible results, as has been done in GWAS throughout its history. The same would be true for human brain imaging studies that explore structural and functional alterations associated with phenotypes in a brain-wide manner [123].

Third, as is true in any field of science, often important unresolved problems, such as the mystery of the molecular brain pathology of schizophrenia, are addressed by the utilization of new technologies. Considering the major limitations of current research on molecular pathology using patient or model brains, while the etiological validity of the model is undoubtedly important, in this context, there is an inherent concern that the molecular pathology in human schizophrenia patients may not be adequately reproduced in rodent or cellular models, even when the exact same variants that are pathogenic in humans are introduced. Perhaps this problem would be addressed by studying species closer to humans, specifically non-human primates. Owing to recent innovations in genome editing technology, genetically engineered non-human primates carrying mutations that are pathogenic in humans, such as cynomolgus monkeys with mutations in *MECP2* [124], the gene responsible for Rett syndrome, or *SHANK3* [125], an ASD gene in the Phelan–McDermid syndrome critical region, and common marmosets with a mutation in *PSEN1* [126] causal for familial Alzheimer’s disease, have been created and analyzed. While research using primates is much more time- and cost-consuming than studies of mice, non-human primate models of schizophrenia will be an excellent resource to fill the gap between humans and rodents. Another major limitation is that while we can comprehensively analyze transcriptomic and epigenomic profiles in postmortem brain tissues, such analysis in the brain of living patients can not be performed. On the other hand, recent technological advances have allowed us to quantify some key molecules involved in the regulation of synapses and histone modifications, such as the synaptic vesicle glycoprotein 2A [127], AMPA-type glutamate receptors [128], and histone deacetylases (HDACs) [129] in the living human brain. By expanding the repertoire of measurable molecules and scaling up studies, we will better understand molecular changes in the brain of living schizophrenic patients.

Besides them, one of the most prominent new technologies that have become prevalent over the last decade is single-cell sequencing techniques, whose usefulness was mentioned in the above section describing the convergent results of cell type enrichment analysis in the PGC GWAS and SCHEMA study. Single-cell analyses are particularly powerful in studies of organs where different cell types are intermingled, such as the brain. By performing cell type-resolved analysis using single-cell technology, it may be possible to more clearly capture molecular pathology that was obscured in bulk tissues. At the end of this section, we highlight pioneering single-cell (nucleus) RNA sequencing studies of postmortem schizophrenia brains, while some of them have been posted to preprint servers and have not been published after peer review.

### Single-nucleus RNA sequencing studies of postmortem schizophrenia brains

Technically, analysis of single “cells”, including cytoplasm, cannot be currently performed in studies of frozen postmortem brain tissues; therefore, single-“nucleus” RNA sequencing (snRNA-seq) studies of human schizophrenia brains have been conducted.

To our knowledge, there are four publications on snRNA-seq of postmortem schizophrenia brains, including two preprints that have not yet been peer-reviewed. Among them, the largest study by Ruzicka et al. analyzed 266,431 nuclei from 24 schizophrenia patients and 293,589 nuclei from 24 controls using frontopolar cortex (Brodmann area 10) samples [130]. In this study, 20 cell types/states were annotated based on their transcriptional profiles, and it was shown that the majority of genes differentially expressed in schizophrenia occurred in the neuronal population. The cell types with the strongest enrichment of schizophrenia-associated genes identified by GWAS among differentially expressed genes include cortico-cortical projection neurons in the deep layers V/VI, parvalbumin-positive basket interneurons, and excitatory neurons of a novel cell state enriched in the supragranular layers II/III. This novel type of supragranular excitatory neurons was more abundant in schizophrenia than in controls, but was preferentially found in schizophrenia individuals with less “transcriptional pathology score”, defined by overall schizophrenia-associated transcriptional dysregulation in each individual. Based on this observation, the authors speculated that this population of excitatory neurons, named Ex-SZTR, might be associated with “schizophrenia transcriptional resilience”. While further scrutinization through peer reviews is needed, this finding may contribute to conceptual advances in the understanding of the molecular/cellular pathology of schizophrenia.

The observation that the majority of differentially expressed genes are found in neuronal populations was also reported in another study by Reiner et al., where 127,930 and 145,120 nuclei from DLPFC of 12 schizophrenia and 14 control individuals were analyzed by snRNA-seq, respectively [131]. In their study, ~96% of differentially expressed genes were observed in neuronal cell types, including excitatory neurons across layers II-V and parvalbumin-positive interneurons.

In a study by Batiuk et al., not only snRNA-seq of sorted neurons from DLPFC of 9 schizophrenia patients and 14 controls (81,817 and 127,236 nuclei, respectively) but also follow-up immunohistochemistry, single-molecule fluorescence in situ hybridization, and spatial transcriptomics analyses in an extended cohort were performed [132]. Results of these analyses convergently suggested that transcriptional dysregulation and altered cellular composition within the upper cortical layer, involving both GABAergic interneurons and principal projection (excitatory in the cortex) neurons, might be a core substrate associated with the brain pathology of schizophrenia.

These results would collectively support that schizophrenia is primarily a disease of neuronal cells. On the other hand, a unique study focusing on cells constituting the blood-brain barrier (BBB) based on the neurovascular hypothesis of schizophrenia was conducted by Puvogel et al. [133]. In their study, a total of 178,009 nuclei (NEUN and OLIG2 negative nuclei enriched for BBB cells and NEUN positive and OLIG2 negative nuclei enriched for neuronal cells) from postmortem midbrain tissues of 15 schizophrenia patients and 14 controls were analyzed by snRNA-seq. The results showed that there was no significant difference in the relative proportions of the major BBB cell types between schizophrenia and controls. A limited number of genes were differentially expressed in schizophrenia (14 genes with log_2_ fold change > 0.3 and FDR < 0.05). These differentially expressed genes were restricted to ependymal cells and pericytes, suggesting that BBB cells are not broadly affected in schizophrenia.

Overall, many of the findings in these studies are detectable only when cell type-resolved analysis is performed, demonstrating the value of snRNA-seq. Nevertheless, some of the above-described results should be considered preliminary because half of the four studies highlighted here have not been peer-reviewed yet. Also, the numbers of individuals analyzed in these studies are not large, while the numbers of nuclei examined were huge. Therefore, it is necessary to consider whether the sample size is sufficient.

## Perspectives: a decade after the best of times, the worst of times for psychiatric disease

In 2012, Karayiorgou et al. on behalf of the Genetic and Neural Complexity in Psychiatry 2011 Working Group described the situation at that time as “the best of times, the worst of times for psychiatric disease” [134]. This was because, on the one hand, the development and deployment of long-awaited new DNA sequencing technology (i.e., next-generation sequencing) made it possible to conduct genome-wide exploration of highly penetrant rare variants on a population scale (the best of times), while on the other hand, many pharmaceutical companies withdrew from the research and development of novel therapeutics due to their low success rates (the worst of the times). A decade later, as predicted, several robust risk genes with large effect sizes have been identified for schizophrenia, and the first results of pioneering studies using animal and cellular models created on the basis of the discovery of these genes are beginning to be harvested [88–92, 106]. Overall, it can be said that we have achieved the expected outcomes over the past ten years. Also, a number of powerful new technologies have been developed and implemented during this period. The important thing is to continue this progress, and such effort will reverse the retreat from research and development by pharmaceutical companies and other investors, which was recognized a decade ago and persists today.

In this context, it would be meaningful to provide a clearer picture of how the field of schizophrenia genetics and biology will further develop. In our view, the overarching challenge for the next decade will be how we translate the findings in basic genetic and biological research into clinical psychiatry. The first part of the path to resolving this problem has been clarified by the results of studies to date. Aggregating the existing knowledge, we are able to identify diagnostic genomic variants (e.g., Pathogenic or Likely Pathogenic variants in the American College of Medical Genetics and Genomics [ACMG] guidelines [135]) in 1–6% of schizophrenia patients by comprehensively analyzing rare variants [136–139], and to extract a small proportion of the population with high genetic risk (e.g., OR > 5) utilizing the overall profiles of common variants (i.e., PRS [140, 141]). On the other hand, to our knowledge, there are no genetic tests for schizophrenia approved by the government and covered by health insurance. Among several reasons for this situation, the primary one is that the clinical benefits gained from genetic testing are far less than the cost and potential side effects. More specifically, there are two major factors limiting the benefits: the performance of risk prediction from genetic information is insufficient, and the results of genetic testing rarely lead to changes in clinical actions. To improve the performance of genetic risk prediction, as described above, it is indispensable to expand the sample size and investigate various types of variants, which include not only common SNPs, rare coding SNVs, and CNVs but also non-coding rare variants, repeat element variants, complex structural variants, somatic variants, and others, with sufficient statistical power. In particular, the variants that are not common but not extremely rare, which can fill the blank region in Fig. 1, will be a major target in future research. Also, it is crucial to conduct sufficiently large studies in diverse ethnic populations. The importance of such studies is evident from the observation that the performance of PRS is greatly reduced when the ethnicity of the individuals being scored is different from that of the data used to construct the prediction model [142, 143]. Regarding the improvement of clinical actionability, it is expected that the generation and investigation of multiple etiologically valid schizophrenia models, as featured in this review, will play an important role. The realization of precision medicine, such as gene therapy, for specific genetic diseases frequently comorbid with schizophrenia (e.g., 22q11.2 deletion or *SETD1A* haploinsufficiency syndrome) leveraging the observations in studies of these models might be the achievable goal within the next decade. And beyond that, by integrating the results of human genetics and model studies as well as other areas of research, such as human functional imaging and brain circuity, it should be aimed to define biologically homogeneous schizophrenia subgroups and identify the optimal treatment and prevention for them.

The World Health Organization estimates that by 2030 mental disorders will be the leading cause of disease burden globally [144], of which a significant part should be accounted for by schizophrenia due to its chronic and often treatment-resistant nature. Studies toward the elucidation of the molecular pathology of schizophrenia, which forms the foundation for essential therapeutics, are of great social value. Therefore, continuous investments from academia, government, industry, and citizens, along with appropriate ethical, legal, and social considerations, are warranted.

### Supplementary information

Supplementary note
