# Supplementary material for: The molecular pathology of schizophrenia: an overview of existing knowledge and new directions for future research
Source: Mol Psychiatry. 2023 Mar 6;28(5):1868–89. doi: 10.1038/s41380-023-02005-2 (PMC10575785; doi:10.1038/s41380-023-02005-2)
Supplement: Supplementary file 1 — Supplementary note [file 41380_2023_2005_MOESM1_ESM.docx]

**Supplementary note**

**The molecular pathology of schizophrenia: an overview of existing knowledge and new directions for future research**

Takumi Nakamura^1^, Ph.D. and Atsushi Takata^1,2*^, M.D., Ph.D.

**Affiliations:**

1. Laboratory for Molecular Pathology of Psychiatric Disorders, RIKEN Center for Brain Science, 2-1 Hirosawa, Wako, Saitama 351-0198, Japan
2. Research Institute for Diseases of Old Age, Juntendo University Graduate School of Medicine, 2-1-1 Hongo, Bunkyo-Ku, Tokyo, 113-8421, Japan

**Running Title:** The molecular pathology of schizophrenia

***Correspondence should be addressed to:**

Atsushi Takata, M.D., Ph.D.

Laboratory for Molecular Pathology of Psychiatric Disorders, RIKEN Center for Brain Science, 2-1 Hirosawa, Wako, Saitama, 351-0198, Japan

Phone & Fax: +81-48-467-9703

Email: atsushi.takata@riken.jp

***Detailed information on other types of variants potentially explaining still-missing heritability***

Although large-scale genetic studies and refinements in statistical methods have elucidated a substantial part of the genetic architecture of schizophrenia, there remains a large gap between the overall heritability reported in epidemiological studies (60-80%) [1,2,3,4] and that explained by common SNPs (24% in ref. [5]) or rare gene-disruptive rare SNVs, indels, and CNVs (< 10% according to refs. [6,7]). It is assumed that at least some of the remaining still-missing heritability is explained by rare variants in non-coding regions that have not yet been fully investigated. Indeed, whole-genome sequencing (WGS) studies of height, body mass index, and type 2 diabetes [8,9] have reported that rare non-coding variants, especially those in low linkage disequilibrium with neighboring variants, explain a substantial part of the unsolved heritability.

*Non-coding variants*

For schizophrenia, a relatively large WGS study analyzing 1,162 cases and 936 controls [10] showed that the heritability explained by all sequencing variants (0.56, standard error = 0.51, n of variants = 17,364,971) is larger than that explained by variants at SNP locations in the HapMap3 reference (0.45, standard error = 0.089, n of variants = 1,189,077). Although larger studies are needed for accurate estimates, this result provides real-world evidence that rare non-coding variants do explain a part of the still-missing heritability of schizophrenia. Also, this WGS study reported enrichment of rare structural variants disrupting boundaries of topologically associated domains (TADs), units of genomic regions where physical interactions between regulatory DNA sequences frequently occur, in schizophrenia cases, further suggesting that genetic variants that affect non-coding regulatory elements contribute to the risk for schizophrenia.

*Tandem repeat variants*

Besides SNVs (including SNPs) and CNVs, tandem repeats variants are another class of genetic variation that have recently gained increasing attention for their contribution to human complex traits, such as autism spectrum disorders (ASD), height, and biomarker levels [11,12,13,14]. In an analysis of tandem repeat expansions using WGS data from 257 schizophrenia cases and 2,729 controls (225 with congenital heart disease and no psychotic illness and 2,504 individuals from the 1000 Genomes Project), an increased burden of near-exon rare tandem repeat expansions in schizophrenia was observed [15]. Although tandem repeat variants are often in linkage disequilibrium with SNPs/SNVs, their contributions were likely not fully captured in previous studies, as often they have more than two alleles and their length can be altered even in one generation. Therefore, tandem repeat variants can explain another important part of the still-missing heritability of schizophrenia, while larger studies and more detailed examination of confounding factors, such as population stratification, are warranted to more accurately estimate their contributions.

*Postzygotic mosaic variants*

As mentioned at the beginning of this review, the DNA sequences of all cells in the body are "in principle" identical; however, this is not always the case. Some genetic variants are generated in a postzygotic stage, and these somatic variants can contribute to a variety of human diseases, including psychiatric disorders [16,17,18,19,20,21]. Strong statistical evidence for an overall contribution of somatic variants in schizophrenia was provided by a study of somatic CNVs in blood-derived DNAs from 12,834 schizophrenia cases and 11,648 controls [22]. In this study, it was demonstrated that somatic CNVs are more frequent in cases than controls (0.91 % vs 0.51%, P = 2.68 × 10^-4^). Another notable observation was that recurrent somatic deletions of exons 1-5 of *NRXN1*, a gene whose germline CNVs are known to be causally associated with schizophrenia and other neuropsychiatric/neurodevelopmental disorders [23], were observed in five schizophrenia cases and not in controls. In addition to this study of blood-derived DNAs, several studies have investigated brain somatic variants, which may be more directly related to the molecular pathology. Among peer-reviewed publications, two high-coverage exome sequencing studies on somatic SNVs in the postmortem dorsolateral prefrontal cortex (DLPFC), each analyzing brain genomes from 58 (27 cases and 31 controls) [24] or 19 (9 cases and 10 controls) individuals [25], were reported. While there is an inconsistency between these two studies in terms of the overall enrichment of deleterious somatic SNVs in schizophrenia, it is notable that somatic missense SNVs were identified in multiple known neurodevelopmental genes, including *GRIN2A*, *EP300*, and *DEPDC5*. Besides these studies, there are preprints reporting results of high-coverage WGS studies analyzing somatic variants throughout the genome [26,27]. In a study of isolated DLPFC neurons from 61 schizophrenia and 25 control individuals, striking enrichment of somatic SNVs in schizophrenia, primarily driven by non-coding mutations especially those in open chromatin transcription factor binding sites, was observed [26]. Another study that examined genomes of 131 human brains (cortex, striatum, or hippocampus) from 59 ASD, 19 Tourette syndrome, 9 schizophrenia, and 44 control individuals [27] reported that there are brains with unusually large numbers of somatic SNVs, termed hypermutable brains. In the context of disease association, enrichment of somatic deletions and SNVs creating putative transcription factor binding motifs in enhancers active in the developing human brain was observed in ASD, collectively supporting the role of somatic variants affecting regulatory elements bound by transcription factors in diseases.

Although somatic variants are in principle not directly explaining heritability, studies of this class of variants may provide important insights into the molecular pathology of schizophrenia, since it is assumed that brain somatic variants preferentially disrupt specific cell types and/or neural circuits, which might be causally associated with the disease pathogenesis.**References**

1. Owen MJ, Sawa A, Mortensen PB. Schizophrenia. Lancet. 2016;388:86-97.

2. Lichtenstein P, Yip BH, Bjork C, Pawitan Y, Cannon TD, Sullivan PF, et al. Common genetic determinants of schizophrenia and bipolar disorder in Swedish families: a population-based study. Lancet. 2009;373:234-9.

3. Sullivan PF, Kendler KS, Neale MC. Schizophrenia as a complex trait: evidence from a meta-analysis of twin studies. Arch Gen Psychiatry. 2003;60:1187-92.

4. Wray NR, Gottesman II. Using summary data from the danish national registers to estimate heritabilities for schizophrenia, bipolar disorder, and major depressive disorder. Front Genet. 2012;3:118.

5. Trubetskoy V, Pardiñas AF, Qi T, Panagiotaropoulou G, Awasthi S, Bigdeli TB, et al. Mapping genomic loci implicates genes and synaptic biology in schizophrenia. Nature. 2022;604:502-8.

6. Visscher PM, Goddard ME, Derks EM, Wray NR. Evidence-based psychiatric genetics, AKA the false dichotomy between common and rare variant hypotheses. Mol Psychiatry. 2012;17:474-85.

7. Gaugler T, Klei L, Sanders SJ, Bodea CA, Goldberg AP, Lee AB, et al. Most genetic risk for autism resides with common variation. Nat Genet. 2014;46:881-5.

8. Wainschtein P, Jain D, Zheng Z, TOPMed Anthropometry Working Group, NHLBI Trans-Omics for Precision Medicine (TOPMed) Consortium, Cupples LA, et al. Assessing the contribution of rare variants to complex trait heritability from whole-genome sequence data. Nat Genet. 2022;54:263-73.

9. Wessel J, Majarian TD, Highland HM, Raghavan S, Szeto MD, Hasbani NR, et al. Rare Non-coding Variation Identified by Large Scale Whole Genome Sequencing Reveals Unexplained Heritability of Type 2 Diabetes. medRxiv. 2020; doi:10.1101/2020.11.13.20221812.

10. Halvorsen M, Huh R, Oskolkov N, Wen J, Netotea S, Giusti-Rodriguez P, et al. Increased burden of ultra-rare structural variants localizing to boundaries of topologically associated domains in schizophrenia. Nat Commun. 2020;11:1842.

11. Mukamel RE, Handsaker RE, Sherman MA, Barton AR, Zheng Y, McCarroll SA, et al. Protein-coding repeat polymorphisms strongly shape diverse human phenotypes. Science. 2021;373:1499-505.

12. Xiao X, Zhang CY, Zhang Z, Hu Z, Li M, Li T. Revisiting tandem repeats in psychiatric disorders from perspectives of genetics, physiology, and brain evolution. Mol Psychiatry. 2022;27:466-75.

13. Trost B, Engchuan W, Nguyen CM, Thiruvahindrapuram B, Dolzhenko E, Backstrom I, et al. Genome-wide detection of tandem DNA repeats that are expanded in autism. Nature. 2020;586:80-6.

14. Mitra I, Huang B, Mousavi N, Ma N, Lamkin M, Yanicky R, et al. Patterns of de novo tandem repeat mutations and their role in autism. Nature. 2021;589:246-50.

15. Mojarad BA, Engchuan W, Trost B, Backstrom I, Yin Y, Thiruvahindrapuram B, et al. Genome-wide tandem repeat expansions contribute to schizophrenia risk. Mol Psychiatry. 2022;27:3692-8.

16. Rodin RE, Dou Y, Kwon M, Sherman MA, D'Gama AM, Doan RN, et al. The landscape of somatic mutation in cerebral cortex of autistic and neurotypical individuals revealed by ultra-deep whole-genome sequencing. Nat Neurosci. 2021;24:176-85.

17. Sherman MA, Rodin RE, Genovese G, Dias C, Barton AR, Mukamel RE, et al. Large mosaic copy number variations confer autism risk. Nat Neurosci. 2021;24:197-203.

18. Lim ET, Uddin M, De Rubeis S, Chan Y, Kamumbu AS, Zhang X, et al. Rates, distribution and implications of postzygotic mosaic mutations in autism spectrum disorder. Nat Neurosci. 2017;20:1217-24.

19. Krupp DR, Barnard RA, Duffourd Y, Evans SA, Mulqueen RM, Bernier R, et al. Exonic Mosaic Mutations Contribute Risk for Autism Spectrum Disorder. Am J Hum Genet. 2017;101:369-90.

20. Nishioka M, Kazuno AA, Nakamura T, Sakai N, Hayama T, Fujii K, et al. Systematic analysis of exonic germline and postzygotic de novo mutations in bipolar disorder. Nat Commun. 2021;12:3750.

21. Nishioka M, Bundo M, Iwamoto K, Kato T. Somatic mutations in the human brain: implications for psychiatric research. Mol Psychiatry. 2019;24:839-56.

22. Maury EA, Sherman MA, Genovese G, Gilgenast TG, Rajarajan P, Flaherty E, et al. Schizophrenia-associated somatic copy number variants from 12,834 cases reveal contribution to risk and recurrent, isoform-specific NRXN1 disruptions. medRxiv. 2022; doi:10.1101/2021.12.24.21268385.

23. Marshall CR, Howrigan DP, Merico D, Thiruvahindrapuram B, Wu W, Greer DS, et al. Contribution of copy number variants to schizophrenia from a genome-wide study of 41,321 subjects. Nat Genet. 2017;49:27-35.

24. Kim MH, Kim IB, Lee J, Cha DH, Park SM, Kim JH, et al. Low-Level Brain Somatic Mutations Are Implicated in Schizophrenia. Biol Psychiatry. 2021;90:35-46.

25. Fullard JF, Charney AW, Voloudakis G, Uzilov AV, Haroutunian V, Roussos P. Assessment of somatic single-nucleotide variation in brain tissue of cases with schizophrenia. Transl Psychiatry. 2019;9:21.

26. Maury EA, Jones A, Seplyarskiy V, Rosenbluh C, Bae T, Wang Y, et al. Enrichment of somatic mutations in schizophrenia brain targets prenatally active transcription factor bindings sites. bioRxiv. 2022; doi:10.1101/2022.02.23.481681.

27. Bae T, Fasching L, Wang Y, Shin JH, Suvakov M, Jang Y, et al. Analysis of somatic mutations in 131 human brains reveals aging-associated hypermutability. Science. 2022;377:511-7.
